# From Regulating Emotions to Less Lonely Screen Time: Parents’ Qualitative Perspectives of the Benefits and Challenges of Adolescent Pet Companionship

**DOI:** 10.3390/bs12050143

**Published:** 2022-05-13

**Authors:** Linda Charmaraman, Stephanie Cobas, Jules Weed, Quan Gu, Elizabeth Kiel, Holly Chin, Alyssa Gramajo, Megan K. Mueller

**Affiliations:** 1 Wellesley Centers for Women, Wellesley College, Wellesley, MA 02481, USA; scobas@wellesley.edu (S.C.); jweed@wellesley.edu (J.W.); qg100@wellesley.edu (Q.G.); ek5@wellesley.edu (E.K.); hchin@wellesley.edu (H.C.); amannin5@wellesley.edu (A.G.); 2 Cummings School of Veterinary Medicine, Tufts University, North Grafton, MA 01536, USA; megan.mueller@tufts.edu

**Keywords:** companion animals, pets, human–animal interaction, adolescent development, parent perspectives, well-being, social-emotional adjustment, qualitative methods

## Abstract

Adolescence is a prime developmental period to explore human–pet relationships, particularly given that teens are often relying less on their families, and more on other attachment figures such as peers and pets. However, most research on pet companionship is conducted with adults and young children. Moreover, lived experiences around having pets in households with adolescents are underexplored, particularly from parents’ perspectives. This qualitative interview study of 31 parents/guardians in the Northeast U.S. explored perceptions of the benefits and challenges of having pets for their adolescent’s well-being as well as how adolescents affected their pet’s well-being. Our three main themes for perceived benefits of pets included social (e.g., reducing anxiety), physical (e.g., screen time companionship), and emotional (e.g., regulation of difficult emotions such as anger, loneliness). Challenges to adolescent well-being included such social topics as family tension around unevenly shared responsibilities, physical themes such as problematic animal behaviors, and emotional themes related to grieving the passing of pets. We offer a developmental systems approach to understanding pets within adolescent families, noting future directions for developing family interventions to improve pet–adolescent interactions given the demands of child and pet upbringing during adolescence.

## 1. Introduction

A majority (70%) of U.S. households [1] currently have a pet, more than half of which are dogs (51.8%) and almost a quarter are cats (23.8%). In recent years, family size in the U.S. has decreased with a reduction in children and elderly in family homes, which may indicate that children are increasingly more likely to grow up with an animal than with a younger sibling or grandparent [2]. Regardless, adults with children (63.8%) are more likely to have pets than adults without children (57.7%) and single adults (42.9%) [3]. Given that 80% of pet owners report that they consider their pets family members [4], it is important to study the benefits and challenges of households with both children and pets. Furthermore, companion animals may play a uniquely important role in the context of the COVID-19 pandemic. Adolescents in particular have experienced significant stressors regarding school, social isolation, and disruption to routines due to school closures and lockdowns, which has the potential to impact the psychosocial well-being of teenagers [5,6]. While the role of pets in youth development continues to be an area of research focus, relatively little is known about family-level dynamics regarding companion animals. In particular, more investigation is needed to understand how parents shape youth experiences with pets and integrate them into the household during the early adolescent years.

### 1.1. Social/Emotional Developmental Needs of Adolescents and the Role of Pets

Early adolescence is a vulnerable age with significant social-emotional challenges associated with lower self-esteem, weaker academic performance, and increased anxiety and competition with others [7], in addition to managing school transitions, peer relationships, and self-regulation skills [8,9]. Therefore, exploring the role of the wider family environment (including pets) in fostering teen resilience is required. As non-judgmental “family” members, pets can help adolescents fulfill two major developmental goals: to feel competent (self-efficacy) and autonomous (independence from parents). Early adolescence (aged 11–14) can include periods of social transition (e.g., school transitions, getting one’s first smartphone, signing up for social media). During this developmental period, social technologies have the potential to dominate the daily lives of adolescents, with 95% of teens aged 13–17 having access to smartphones and 45% going online constantly [10]. Notably, in May 2020, adolescents aged 10 to 14 spent, on average, 7.70 h per day on screens, compared to a pre-pandemic estimate of 3.8 h per day [11]. Strong social relationships outside of the school setting can provide a buffer for some of the social-emotional effects of these transitory periods [8].

One previous study observed parallels between children’s interaction patterns with animals and with peers, suggesting that strong connections with pets may relate to better adjustment socially [12]. Pets provide stable social contact, positive social interactions, and social facilitation that may contribute to resilience in the broader context of an adolescent’s social context. These findings suggest that adolescence is a prime developmental period for exploring human–pet relationships, particularly given that in this stage, teens are moving away from relying on their parents and siblings and focusing more on other attachment figures such as peers, romantic partners, and pets. For instance, 12-year-olds are spending more time caring for pets than caring for younger siblings, when both are present in their families [13]. Therefore, understanding the nuances of how pets fit into the social support network is important in fully exploring adolescent health.

Pets may offer adolescents solace from strained familial relationships in this period of particular emotional turbulence [14]. Research in Australia has shown that children with reduced access to parental resources have stronger bonds with their dogs [15]. Pets’ potential to act as confidants is critical, as early adolescence is associated with high self-consciousness [16] and low self-disclosure [17]. More than 70% of young people report confiding in a pet [18] since they do not have to worry about judgment or needing to meet expectations [19]. A recent review found that pet ownership—particularly *dog* ownership due to higher levels of interaction and reciprocation compared to other pets—has beneficial effects on cognitive, socio-emotional, and behavioral development [20]. Adolescents with dogs demonstrated higher levels of pet attachment and, in turn, showed higher levels of overall life satisfaction [14]. However, despite the potential for pets to contribute positively to adolescent well-being, existing research also includes a number of studies that find no positive associations between companion animals and youth mental health [20,21,22]. Furthermore, the relationship quality between youth and their companion animals can be a key factor in driving any potential benefits of pet ownership. Therefore, there is a need for research that explores the nuances of these relationships to assess more precisely the role of pets in adolescent development.

### 1.2. Parent Perspectives Regarding Role of Pets

The vast majority of pet–children interaction studies to date have focused on children’s perspectives regarding attachment to their pet companions. Much less is known about parent perceptions of how the family pet will play a key role in their child’s social, emotional, and physical development, particularly in the adolescent years. Fifield and Forsyth [23] found that parents of children aged 8–12 most often cited teaching responsibility, the child’s desire to have a pet, and companionship for the child as the main reasons for initially acquiring a pet. In the same study, parents claimed that the primary advantage of having a pet was teaching their children to be responsible and caring. One of the rare studies of parent attitudes toward pet ownership asked 50 parents about their reasons for having pets in the family specifically for their child’s development [24]. Their responses included providing an unconditional playmate and listener, opportunities to develop a sense of responsibility, respect for animals, and natural observations of life processes. This is consistent with Jalongo and Ross’s [25] recent finding of three thematic trends in the literature on the topic of parents’ motivations to introduce dogs into the family: to please children, to provide children companions to grow up with, and to teach children responsibility. A body of research on adult pet ownership shows that pets serve as a catalyst for social interaction [1], and pet ownership is associated with higher levels of social capital [2] and physical activity [3,4]; however, this same research does not exist for adolescents who own a pet.

Other studies demonstrate that pets sometimes offer a way to triangulate a strained family system, in that a child can be overheard saying something to a pet and a parent receives the message [26]. For instance, one study [27] documented that within dog-owning families, parents and children used dogs to “frame shift” a conversation, such as when buffering criticisms or teaching values. With prior research on parental attitudes more focused on the social and emotional benefits of having pets, less is known about how parents view the potential physical benefits of petting behaviors, such as the stress-buffering role of socially touching pets, which was found to bring comfort to children [28].

Prior research has found a wide range of attitudes concerning household pets, and that pet ownership is not uniformly beneficial for all families. Many pet owners feel that pets are family members and that animals can communicate and feel empathy toward them in times of distress [29]. However, some pet owners, as well as non-pet owners, perceive animals as a burden, an unwelcome responsibility, untidy, a strain on finances, competition for time with social and career commitments, and a potential source of conflict with family and neighbors [30]. Caring for a sick pet can also cause a significant caregiver burden for owners, contributing to stress and anxiety [31]. A recent study of pet owners [32] found that 56% of respondents expressed some type of pet-related challenge or concern related to or exacerbated by the COVID-19 pandemic. The study observed that pet owners experience different types of pet-related concerns, correlated with the strength of attachment to their pet, their financial situation, and their household makeup. Little is known about parental attitudes regarding the challenges of having pets specifically in the adolescent years.

### 1.3. Effects of Youth–Animal Interactions on Pet Wellbeing

Very little is known about the impact that child–pet interactions, especially in families with adolescents, may have on pet well-being. However, a recent study found that pet owners perceived their pets’ well-being to be improved during lockdowns associated with COVID-19 in New Zealand [33]. The participants in the study cited increased human–pet interaction and interaction with social media content created by animal experts. This study suggests that perhaps higher levels of interactions with companion animals could provide some benefit for pets via companionship and enrichment. Another recent study on the effects of COVID-19-related lockdowns in the UK found that although dogs were reported to help their owners cope with feelings of loneliness, many shared concerns about their pet’s ability to cope with “alone time” and saw an increase in destructive and attention-seeking behavior [34]. A similar study of a US sample showed similar concerns arising: a quarter of pet owners expressed concerns about meeting their pets’ social and behavioral needs, while smaller percentages of respondents faced issues of access to pet supplies and veterinary care as a result of lockdowns [32]. A retroactive study of pediatric dog bites during COVID-19 lockdown in an Italian city concluded that pandemic stressors on dogs, including disrupted exercise and more time spent with families, could explain the significant increase in young children being bit by family dogs, suggesting that lockdowns had negative effects on youth and pets alike [35]. However, the exact role of specific types of child–pet interactions on animal well-being has been significantly under-explored in the human–animal interaction (HAI) literature. Parents may be uniquely suited to observing their adolescents’ interactions with their companion animals in real-life settings.

Some research has found that the strength of relationships, preparedness for ownership, and the presence of pets may dictate how well youth will treat their pet and perceive their well-being. Having a good relationship with a pet can be critical for the pet’s well-being, as Hawkins et al. [36] found that youths with a strong attachment to a pet showed more humane treatment of animals and had a greater concern for animal welfare. However, children that were ill-prepared to have a pet, notably toddlers, are more likely to harm pets by chasing, collapsing, stepping on or squeezing animals without considering the effect it may have on them [37].

Some evidence suggests that specific home environments can also have a negative effect on pet well-being. For example, studies show a link between domestic violence and animal cruelty in homes [38] due to the “shared situational characteristics common to a range of violent behaviors” [39]. Caring for a pet can also be associated with stressors, such as caregiver burden [31], as well as higher than expected financial costs [40]. However, the impact of these challenges on the animals themselves has not been studied in detail. Recognizing that youth–pet relationships are mutually influential, a dynamic relationship is critical. Understanding how different family dynamics may contribute to health and well-being for both humans and animals optimizes adolescent–pet relationships.

### 1.4. Developmental Systems Approach to Understanding Pets in the Family

In order to understand the nuances and complexities of youth–companion animal relationships and how they may contribute to adolescent development, a systems-based theoretical approach that allows for exploration of the integrated contexts that contribute to human–animal interaction must be considered. Relational developmental systems theories [41] provide a useful framework for assessing these relationships. This framework views youth development as the product of mutually influential, dynamic relationships between an individual and the many integrated layers of a youth’s ecosystem [42]. Within this framing, youth–pet relationships do not exist in isolation; they influence and are influenced by the many other aspects of the ecosystem. Understanding how companion animals fit into this ecosystem is necessary to more fully assess the complexities and variations in how youth–pet interactions can contribute to both human and animal health and well-being. For example, pet owners often view their pets as members of the family [26,30] and can become greatly attached to them, treating them as if animals can communicate and can reciprocate in empathy [26,43]. Therefore, the way in which parents, directly and indirectly, socialize pets into the family dynamic may have a strong influence on a child’s “construction” of companion animals, and can therefore mediate health outcomes associated with the child–pet relationship. Within this dynamic systems perspective on child development [43], children’s diverse relationships (within the home, school, neighborhood, etc.) are nested within and mutually affect one another [44]. Parents can provide a unique perspective on how youth–pet relationships intersect with these other aspects of their child’s developmental system.

### 1.5. Current Study

Despite the presence of pets in the majority of households with children in the US and the importance of parents in shaping youth–pet interactions within the family, there is relatively little research exploring the benefits and challenges of having pet companions as parents navigate the complexities of raising both humans and animals together. Prior research has focused on more quantitative designs [20] and intervention research on the impacts of therapy animals on children or adults. Rarely do studies focus on the family system in order to understand the decision-making and observations about a pet’s role in adolescent development from the parent/guardians’ point of view. Therefore, the goal of this study was to assess the following research questions:

1. From the parents’ point of view, how is having a pet beneficial to adolescent development?
2. What are the challenges of having a pet during adolescence?
3. Are there ways in which parents perceive adolescents and/or their families as being beneficial or detrimental to a pet’s well-being?

## 2. Materials and Methods

### 2.1. Participants and Procedures

Drawing on an ongoing mixed-method study of secondary school students’ social technology use in multiple Northeast United States school districts (e.g., masked for review), we invited parents from these schools to participate in an online survey about parental monitoring of digital media use. After obtaining IRB approval from Brandeis University, study disclosures and Qualtrics survey links were distributed through school-partnered contact lists, parent listservs, and e-newsletters from school administrative program coordinators in 2020. All parents of middle school students were eligible to participate in a 20–30-min survey and entered into a raffle for multiple USD 25 gift cards. Surveys were available in English, Spanish, and Portuguese, with consent solicited on the first page.

#### 2.1.1. Survey Sample and Interview Recruitment

A total of 309 parents participated in the online survey. In total, 89% identified as the mother or female guardian of their child, and 49% reported on their daughter. The majority identified as White (55%), followed by 9% identifying as Hispanic, 4% Black, 2% Asian American Pacific Islander, less than 1.0% Multiracial, and 30% of parents did not provide an answer. Parents came from predominantly higher household income brackets with a range of USD 35,000 to USD 200,000+ annually, with an estimated average of USD 124,400 for those that reported salary information. In total, 65% of participants reported an income over USD 100,000, 19% reported an income below USD 100,000, and 16% preferred not to answer. In total, 16% were eligible for free/reduced-price lunch, which is a federal program in the US that allows low-income and/or food-insecure families to feed their children without financial burden. In total, 19% of participants did not complete college. At the completion of the survey, parents were asked if they were interested in a follow-up interview study. From this interview-interest list, our inclusion criteria involved both low and high monitoring parental practices which we determined through the parent survey screener. We contacted 69 parents via direct email in addition to school-wide principal email announcements and parent referrals resulting in 31 interviews. Study disclosures and signed consent were obtained prior to scheduling the interviews. Each participant received a USD 40 gift card for being interviewed. Our team conducted 31 interviews with parents of adolescents from our larger survey study via phone or Zoom to illustrate the family-level processes involved in pet companions during the middle school years

#### 2.1.2. Interview Sample

Interviews were completed with 28 mothers/female guardians, 2 fathers/male guardians, and 1 non-binary parent/guardian. Their demographic data were primarily determined during the screener survey and verified during the interview. Seventy-four percent of the sample owned dogs, 23% owned cats, and 3% owned a guinea pig. We asked parent/guardians to focus their answers on the child/ren who were in middle school. More than half of the parent/guardians referred to daughters (*n* = 21) and 10 referred to sons during their interviews. The gender of the child was determined through either child self-report from the larger ongoing study and/or through the parent determination during the interview. In terms of racial-ethnic composition, 21 parent/guardians (referred to in the rest of the paper as “parents” for brevity) were White (68%), 2 Hispanic (6.5%), 2 Brazilian (6.5%), 1 Black (3%), 1 multiracial/ multiethnic (3%), and 4 unknown (13%).

#### 2.1.3. Interview Protocol

During the hour-long exploratory interview, parents were asked about the role of family pets in the household. Parents discussed their child’s emotional attachment, coping and stress management techniques, caretaking responsibilities, and social media and technology use as it relates to their family household pet. If they had more than one pet, they were asked to focus on their child’s favorite pet. We also asked about the impact of having and/or acquiring a pet during the COVID-19 pandemic, especially during the remote learning period.

### 2.2. Data Analysis

Recorded interviews were transcribed verbatim, verified by the research team, and imported into NVivo software (NVivo 12 Version: Plus). An initial open coding phase by 5 co-authors was used to categorize, sort, and code. Initial nodes were created a priori according to the main interview questions. New codes and subcodes were then created through both deductive and inductive processes. Discussions with team members about reactions to the interview content helped us refine the primary codes, develop secondary codes, and identify any unexpected emerging themes. Our group process of reflexive thematic analysis [45] confirmed that the themes and subthemes were categorized and defined accurately, which aimed to classify higher-order themes that provided meaning to connect lower-level codes. In the final stages, the relationship between aims, research questions, and themes helped determine the theme and subtheme salience [45], thus we calculated theme frequencies to provide transparency in conveying the proportion of participants who reported such experiences and attitudes. A final audit was performed to prevent unintentional over-emphasis of quotes from the same set of parents in the final narrative in order to increase the inclusivity of experiences in our sample. We used pseudonyms for all names of parents, adolescents, and pets to protect their privacy.

## 3. Results

In the next sections, we present the perceived benefits and challenges of pet influences on adolescents, followed by the perceived adolescent family effects on pets.

### 3.1. Benefits to Adolescents

Overall, we found three main themes that encompass the benefits and challenges of having a pet in a household with adolescents: (1) social (30 out of 31 parents), (2) physical (13 out of 31 parents), (3) emotional (30 out of 31) (refer to Table 1 below).

#### 3.1.1. Social Benefits

*Pet as a companion.* Pets were described as bringing social benefits to both adolescents themselves and the family system. Most of the parents (26 out of 31) described pets as a companion for adolescents. In some cases, pets provided them with a means to show affection (without feeling “judged or awkward”) and express positive emotions without feeling self-conscious around others. Maryellen spoke about how her seventh-grade daughter was able to overcome her quiet shyness when others started a conversation about her dog Bella: “It’s forcing her to have a conversation back, and you know she’s that bridge because if Bella wasn’t there, they would say hi but then move on”. John observed that his eighth-grade daughter had personality changes feeling “pretty isolated due to the [COVID-19] pandemic”. He recalled fondly,

If she noticed you she’ll stop, but sneaking around the corner I’ll catch her hanging out with Luna, petting her, snuggling with her…you know, as somebody that doesn’t really know much about it, I think it’s probably positive for to be able to express those positive things in ways that she’s not comfortable with people.

Pets were also described as helping to bring someone “out of her shell,” particularly when trying to meet new people or feeling comfortable talking to people. Teresa’s sixth-grade daughter would invite Max to come and sit with her during remote learning:

…Even though Max is not on the Zoom or whatnot just having her around whether it’s like sitting there on the couch next to her or at her feet will sometimes make Mary feel more comfortable or give her that extra comfort.

*Learning about responsibility.* Over half of the parents interviewed pointed out that pets benefit adolescents by teaching them about independence and responsibility through the process of caretaking. Fiona spoke of how her daughter would pet her guinea pig when she sensed it was stressed. Another parent, Carol, remarked on how her eighth-grade daughter enjoyed caring for their cat by giving shower baths. Marie likened the relationship between her eighth-grade son and their dog to that between “a dad [and] a child” and contended that by “cleaning up and taking care of him” he modeled “being a responsible citizen”. Brenda also spoke positively about how her seventh-grade daughter liked to provide various forms of care to her dog:

So Ava brushes her teeth, sometimes she cleans her ‘butt’. Molly has a little corner where we keep her stuff. So, we have hair bows, brushes, everything. It’s really like she’s her little daughter, you know? Change the water, put her food. It’s a very beautiful relationship to see.

*Building empathy skills.* Many parents (24 out of 31) linked having a pet to adolescents developing empathy towards both animals and humans. When parents reported that their children developed empathy for other animals as a result of the relationship with their pet, it was mostly about an awareness that animals feel physical pain. Jack explains how interactions with the family’s dogs offer a space to teach about empathy:

I mean we have that conversation like every day…someone will walk past and bump the dog or hit the dog. We’ll be like, “Charlie can feel that! How would that feel if somebody hit you? Charlie’s a dog, he can feel you”, you know so like we do some really explicit teaching about empathy.

Developing empathy for other humans was related mostly to other pet owners and family members who interact with their pets. Jane touched on the impact of pet ownership on her adolescent’s empathy toward pet owners who lose a pet:

I think they definitely have an understanding that you know if something happens to a pet it’s not just a pet it’s a family member… our neighbor had to put her pet down and they were like devastated for this family… they were like I can’t imagine that happening to us.

Some parents say that having a pet did not help their children develop empathy. According to one mother, Fiona, her children “would be empathetic anyway” without pet guinea pigs in their lives.

*Bonding with family and friends offline.* More than half (17 out of 31) of parents reflected that pets help families and friends to bond. John’s daughter learned to use a shared love of pets to overcome her “uncomfortable feeling around people”: “If she’s out, and somebody’s got a puppy, she’s very excited to go play with it and pet it, and I think that’s a way to connect with other people around their pets”. Some parents mentioned pets serving as a connection between siblings, and an incentive for adolescents to get out of the room and spend time with family. Dee said that their dog helps her sixth-grade twin daughters to “bond with each other… because they may be twins but they’re polar opposite” and again likened their bond to the pet to a familial relationship: “they’re the big sisters to this little furry brother”. One parent, Leah, also mentioned that pets help her “not super socially adept” sixth-grade son, James, connect with his peers: “socially it helps him because you know he has like a story to tell about the dog or a story to tell about the cat and that gives them a point of connection”. Talking to others about their pets or general love of dogs helped adolescents overcome shyness and social anxiety in order to form connections with others.

*Bonding through socialmedia.* Many (17 out of 31) parents reported that their adolescents post pictures of their pet(s) on social media or through online messaging services with a substantial number (9 out of 17) specifically mentioning the benefit this online sharing plays in maintaining bonds with family and friends. Rachel’s ten-year-old daughter, Catherine, coped with the difficulties of online learning by finding joy in “hugging and kissing and picking up, and [making] Tik Tok videos” with their cat Hazel. The creation of pet-centric media posts also, often, catalyzed positive virtual interactions with their peers, further contributing to the adolescent’s overall social and emotional well-being.

During the pandemic, some parents noted the heightened importance of pet-centric social media posts in fostering connections online. Jennifer’s thirteen-year-old daughter, Lucy, had “photoshoots” of their dog, Stella, which she was “super excited to show her friends” online while the COVID-19 pandemic prevented them from interacting in person. Dee’s twelve-year-old daughter, Gigi, used photos of their dog, Pal, as a conversation starter to remotely rekindle connections with friends:

It’s like the bonding with friends, so I see her taking… tons and tons and tons of pictures of him… if you haven’t talked to someone in a while—like what I see happening—is she’ll have kind of distance between time she’s talked with people and then she’ll take Pal and use him to instigate conversation like, “hey look what, how weird Pal looks today”, because that kind of thing, so... kind of as an entry into social discussions, I guess…

Other adolescents primarily use messaging apps to virtually discuss their pets and foster social connections. Fiona’s thirteen-year-old daughter, Daisy, “will definitely show [her friends] what the animals are up to or take pictures and send them to her friends”, despite not using social media apps. Similarly, Cindy’s daughter, Lily, opts, instead of using social media, to “share some pictures sometimes with her friends, but more [by] texting” although she does maintain an Instagram account as well.

#### 3.1.2. Physical Benefits

Dogs’ presence in a child’s life provides numerous benefits, including an anecdotal increase in physical exercise, reduced screen time, and an increased sense of safety.

*Physical activity displacing screen time.* A third (9 out of 31) of parents remarked how dogs served as an alternative to digital devices during free time, i.e., going on a walk with the family pet rather than watching television or playing video games. Brook, for example, shares,

…especially in COVID, the nice thing about having an animal is that it forces you to actually have to get outside. [Her son is] a typical middle school boy—he could literally be on his gaming systems from sunup to sundown. [He’ll] take her for a mile walk … so it almost forces them to actually have to exercise, which is nice.

Erin remarks similarly about her twin daughters in sixth-grade: “I’ll say put the electronics away, and they’ll say, ‘Oh, but there’s nothing else to do.’ [So,] let’s go do something with the dog, let’s bring her outside…for a walk to the park”.

*Bonding with family during exercise.* Some parents (6 out of 31) claimed exercising as a family helps with family bonding. Jennifer shares how family walks are “another opportunity to get out and have fun with the dog, as well as just a simple run outside during your school break and play some fetch”. Likewise, Brenda also shares how their dog specifically allows for more time together as a family. The mother and her seventh-grade daughter would walk past the father’s workplace, and he would join for the duration of the dog’s walk, allowing for a moment of family togetherness during the workday that might not otherwise be taken. Interestingly, we also found evidence that parents’ perceived interactions with pets can be shaped by age and their gender identity and/or socialization. In the case of one family that had an older son (age 16) and younger twin daughters (both age 12). Dee describes how “it’s a slightly different relationship. My older son uses him as an excuse to get out of the house, he’ll go running with them, exercise with them, whereas the girls... literally will [kiss] him. It’s really gross … it’s much more affectionate”. She describes the girls’ relationship with the pet as more “nurturing” and the son’s as playing “tug of war and rougher stuff with him”. Thus, we see how relationships with the same pet within the same family can provide diverse physical benefits (source of affection, playmate, excuse to exercise) due to a variety of factors.

*Sense of physical/psychological safety.* A few (3 out of 31) parents highlighted the sense of safety, both physically and psychologically, that pets bring to the household. A parent highlighted the sense of safety their child felt in hearing their dog bark, in instances of feeling physically unsafe (i.e., fear of a stalker or robbery). In that time, Jack states, “having the dogs felt important. For all of us, but you know, like anytime that [Johnathan, seventh-grade] is kind of on edge, he hears the dogs barking, he’s like, [who’s here], you know?” Other parents similarly cite a sense of safety with pets, even when they are physically small -- there is greater peace in leaving their children home alone. Maryellen shares about her family’s dog: “he’s super small [and] he won’t save their lives if something really happened. But I know they feel better because he’s around and they can be with him”. Thus, pets’ presences are reassuring to parents and children alike.

Cindy spoke about the role the family dog played in fostering her eighth-grade daughter’s sense of independence:

She loves that she can take him for a walk…so that’s kind of like her independent time too, that she goes outside by herself with the dog…and she feels safer, you know like she has a purpose to go outside.

We see once again that a sense of physical safety and symbol of being home can appear through the presence of a pet, and may be similar to instances of emotional benefits to owning pets.

#### 3.1.3. Emotional Benefits

*Managing social anxiety.* Some parents (8 out of 31) viewed dogs as supportive of their adolescent’s abilities to learn adaptive coping mechanisms, such as to calm down during periods of increased anxiety such as during Zoom classes, while doing homework, or when adolescents are simply in an anxious state of mind. Brooke explained that her family’s emotional support dog understands “when and who needs her” and helps her son eighth-grade son manage his ADHD and anxiety symptoms:

When he’s nervous he’s able to actually have a conversation with a teacher via Zoom or anything like that, just because he’s sitting there petting her. And he’s far more likely to sit still a little bit better um he likes to use rain a lot during the day for school.

Dee discussed the ways that their dog provides a sense of comfort and understanding to her daughter who experiences social anxiety:

One of my middle schoolers she has a lot of anxiety and [the dog] really, really kind of gets that and is the one who really can change her mood better than a [human can], he’s not the most sensitive dog I’ve ever owned or known about, but he kind of, with her in particular, has a special bond and so I’m really pleased with that because he really knows how to snap her out of a mood and I don’t mean obviously, I don’t know if it’s intentional or not, on his part but that has been a very pleasant surprise.

*Regulating emotionsduring digital media use.* The majority of parents (27 out of 31) reported their pets’ presence during media-based family activities like watching television and individual activities like playing video games and using social media. Further, the role that pets play in providing social and emotional support to adolescents may be evolving alongside increased media and technology exposure. While many parents reported the presence of pets during positive interactions with media, like watching television as a family, some discussed the importance of pets following unpleasant media subjection. Jessie reported that her thirteen-year-old daughter, Grace, finds emotional comfort in their dog, Piper, after watching “sad” television programs. Other adolescents find emotional comfort in creating pet-centric social media posts. Beth’s tenth-grade son, Noah, takes “100 photos a day” of their cat, Oliver, noting the importance of entertainment and joy during what seems “like Groundhog’s day, every day”.

*Emotional regulation/coping offline.* More than half (17 out of 31) of parents described their pets as a companion for their adolescents to help with adolescents’ emotional regulation such as anger, grief, and loneliness. Anthony recalls times when their dog would act as a buffer when there were heated arguments within their family:

I have witnessed before when Clara and her sister are arguing, Blade who was the protector of the House, he got in between them and looked at one, looked at the other and they just started to calm down and get kind of quiet and he sat down and kind of waited for them to stop raising their voices and talk like they should have been to each other. And they ended up working out the difference and, once their voices got calm he laid down and once they’re finished he got up and he went off.

Maryellen shared that their dog was the “go to” for her daughter when coping with “little blips with friends: “I mean, when you pet a dog, your blood pressure lowers instantly. And my kids don’t know that, but I think they know what just like patting, I think they don’t really understand, but they must feel it”. Another parent, Jack, said that his seventh-grade son who had previously been in the foster care system had “some big boundaries up” and the family incorporated their dog into teaching him about coping strategies:

He talks to the dog sometimes when he doesn’t talk to people. He doesn’t—it’s hard for him too—he’s got some like big boundaries up. And he’s able to actually literally talk to the dogs. Pet the dog, we’ve talked really explicitly about, calm down strategies like instead of punching the wall, what can we do instead? Like actually petting a dog will bring your heart rate down and that actually helps you, so we’ve been really explicit about strategies and how that might be a good one.

In these cases, the physical touching of their pets were ways to ameliorate intense emotional surges, resulting in calming down the heart rate and becoming grounded again.

Katie shared that her sixth-grade daughter benefitted from playing with their dog after the loss of a family member to COVID-19: “it’s not that they didn’t process maybe what was a little bit challenging or upsetting, but they were able to then kind of respond and say, okay, this is managed, now moving on to something that’s happier and makes me feel better”.

Of course, although most of our examples include dogs, this is not the only pet that may provide comfort through their physical presence. In one case, Beth shares how her son’s cat calls when he arrives home, providing a physical sense of home. She also referenced how he cuddles with the cat when he arrives home from school:

When he was little, it was always difficult—he’d come home from school, never happy. So he comes home, grabs Oliver, and they would just sit on the couch and he just be like talking to the cat and petting the cat and spending time… still he’s 16 now and he comes home and he’s like, ‘Where’s my cat? I need my cat’ and just cuddles with him and it’s nice to see.

### 3.2. Challenges to Adolescent Wellbeing

Similar to the range of beneficial aspects of having pets, we found that parents reported social (16 out of 31), physical (13 out of 31), and emotional (16 out of 31) challenges. Refer to Table 1 above. A small number of parents (5 out of 31) reported not experiencing challenges raising pets.

#### 3.2.1. Social Challenges

*Pet as a distraction.* Some parents (5 out of 31) expressed concerns about the role that pets play in the household and struggled with boundary setting when spending time with pets. For instance, Erin mentioned that in the case of her twin sixth-grade daughters, pets were a constant distraction from what they should be doing (e.g., getting ready for school):

There are times, where I have to say, you have to just leave the dog alone, because I do think the dog can be a distraction, at times, and it’s not the dog’s fault. You guys have to eat breakfast, you need to get on the bus but we’re too concentrated on, Tell the dog good morning, or playing with the dog where they’re not focusing on what they should be doing to start the day.

Similarly, Brenda expressed concern that spending time with the dog was interfering with her homework as if it were an unconscious procrastination method:

So, she wants to be with Molly all the time. And I say to her: “Daughter, you can’t. Now it’s homework, she can’t be on top of you”. “Mom, but she’s so quiet”. “I know she’s quiet but she distracts you from what you’re doing”, and then she puts her on the floor.

While pets, at times, supported adolescents through the challenges of remote learning, they also may act as a distraction in the virtual classroom. A majority of parents (19 of 31) reported that pets were present during their adolescent’s remote learning, while fewer (8 of 31) reported that pets were not present. Lee’s children, including her twelve-year-old daughter, Michelle, were highly attached to their cats. Though she reported that the children were not struggling immensely with online school, she believed that bringing the cats to online school can “sometimes get distracting”. Other parents of adolescents found that pets sometimes interfered more directly with their child’s online work by manipulating their devices in disruptive and erroneous ways. Laura’s thirteen-year-old son, Storm, found that during virtual class, their cat, Nala, “likes to be in front of him, right in front of the camera… a few times she pushed buttons, ‘cause she walks right on [the keyboard]”.

*Family tension over shared responsibility.* Some (8 out of 31) parents struggled to teach the value of sharing pet responsibilities among family members. John, the father of an eighth-grade daughter, expressed his desire for an environment geared towards “connection” and “mutual responsibility” for children, rather than just “hanging out” with their dog: “I think we could have done a better job with it…definitely assigning those responsibilities and trying to make them more structured”. One parent, Jack, explained how their son was happy to complete tasks like feeding and walking but refused to clean up poop for vomit. Katie confessed that it was difficult to motivate her sixth-grade daughter to take care of their dog who is also dealing with their own frustrations:

So yeah, so that’s usually when that comes up when [the kids] just sort of tired and had a long day with the dog already, and, you know, it’s important to know too that [when puppies] turn nine months, they turn into adolescence, so they regress in their behaviors.

Parents talked about chore charts that supported the consistency of caretaking. However, sometimes the push for caretaking and physical support came from pets. Brooke explained how pets may signal a desire for physical support and affection that appears to be a demand rather than a request to their child. For instance, they remarked that their pet would “usually sit on [them] so that [they] actually [couldn’t] get up so they’re forced to do nothing but pet” their animal.

*Jealousy.* A few parents shared difficulties with having pets within the family system caused by jealousy (5 out of 31). Erin claimed that her sixth-grade twin daughters would test the dog’s loyalty by making her choose whom she prefers, creating some jealous tension:

I think that Casey definitely has probably the better relationship? I think sometimes Shay can get a little jealous like they’ll play the game, you know, they’ll both stand on either side of the room, who is the dog going to go to. Most of the time, the dog’s going to go to Casey. But I do have to say that Casey has always been the one that gets up in the morning feeds, checks on her. Shay tends to sleep in late…Casey is very punctual, Casey is very scheduled routine, where Shay is kind of all over the place.

Jessie mentioned the fact that her eighth-grade daughter often implied that her mom was granting their pet more privileges than she was given, such as bedtime curfews, as if the pet was a sibling rival:

The jealousy piece is not something I was anticipating, but I also think she sort of plays with it, to sort of play with our emotions a little bit. When she’ll say like, why does Piper get that and I don’t, or why can Piper stay up and I can’t, like, you know she’ll sort of play with it, I say play with it because I don’t think it’s true…but I’m sure there is a sense of some underlying layer of jealousy, because I do spend quite a bit of time with them, and I enjoy it and I think everybody in the house knows that, and I haven’t had pets in 25 years so it’s nice to have that.

*Screens pulling attention away from pet.* Just as animals can be a popular topic that adolescents consume on their screens on a daily basis, one parent claimed the time spent on screens can also pull them away from paying enough to their pets in real life:

Yeah, they could be on their phone, playing Xbox, chatting with a friend, or just wanting to just sit, you know, like sometimes my daughter will say ‘I was playing with him all day long, why couldn’t he go play with him’, you know. I’m like we’re talking about playing with the dog, I’m not telling you to take out the trash! (Katie about her sixth-grade daughter)

#### 3.2.2. Physical Challenges

*Pets growing bigger.* Katie expressed that her daughter was not willing to take on the responsibility of playing with their growing dog: “So they regress into that early puppy stuff, but the difference is that, you know, he’s not 13 pounds anymore, he is 75 pounds, so trying to manage him can be a bit of a challenge. Yeah, he’s big”.

*Travel restrictions.* Typical in households with dogs, a few (5 out of 31) parents claimed that they encountered travel restrictions. Cindy pointed out that the family was restricted in leaving the house for too long:

Before we used to be out of the house, maybe from six to eight hours, but that is something we have to think about you know, he’s home and we need to go and let him out and feed him and so one more thing in our schedule arrangements, that part, I don’t like that.

Likewise, Jessie described how difficult it was to leave the house when nursing a pet with special caretaking needs:

They become extremely protective of us, which is natural, but it’s made them anxious around other people. Some of us might just be very particular to each dog, but like we would even go on a car ride somewhere, like one of them vomits, it just makes it miserable -we can’t go anywhere. So our life is pretty much like at the house with the dogs.

*Problematic animal behaviors.* A third of the parents (11 out of 31) expressed that the challenge stems from the dogs themselves: either the dog had emotional challenges themselves or families would develop fear and distrust in reaction to aggressive behavior. Jessie, the mother of an eighth-grade daughter, mentions how their dogs are “anxious around other people”. Even going on a car ride may result in vomiting and “misery”, meaning that “our life is pretty much like at the house with the dogs” and “we can’t go anywhere”. The family is unable to travel far or long because of their pet’s well-being.

Parents also identified various causes of aggressive behavior: a lack of or failed training, the breed of the dog, or a possible history of abuse. Amy, a parent with an eighth-grade daughter, for instance, spoke on how a dog they eventually rehomed due to aggression, biting, poor training, and choosing “the wrong breed”. Although the pet is now with “someone that hunts and has two other dogs of the same breed [which may be] for the best… In the end, it ripped out all the good things that he was in the beginning, because she saw it biting me. And I think that was very traumatic to her”. Unfortunately, multiple parents spoke on instances regarding biting of the parent, child, and/or other family members, which has led to trauma on the part of children who were often witnesses or victims to the incident. This has at times led to rehoming of the pets for caretakers suited for the breed and/or training needs, or continuation of their training within the household, with one parent, Jack, who has a seventh-grade son, remarking that “there is a little bit of concern there”, following a biting incident where no blood was drawn, “but I think it’s getting better”. Parents bore the responsibility for training failure, while parents who raised concerns about breed or abuse placed the fault in the animal.

#### 3.2.3. Emotional Challenges

*Financial burden.* Additionally, some parents (4 out of 31) shared their concern for the obligation for raising pets, such as the financial burdens and the shared responsibility for pet caretaking within the household. The eighth-grade daughter of one parent, Jane, remarked, “I don’t think they [prospective pet owners] realize like you gotta make sure that you are financially stable too… We know Hank is going to cost us about $400 [yearly] for vet bills. I think that that’s something that also needs to be a consideration before you choose a pet, like make sure that you’re able to actually take care of this pet the way that they deserve”. Although adolescents are typically not responsible for the financial burden of pet ownership, the costs of companion animals were clearly salient for parents.

*Overly attached.* Contrary to the idea that pets are an extension of one’s family, a few (4 out of 31) parents were concerned that their children had become too emotionally invested in the pet’s role in the family. Amy claimed that she has to caution everyone that “a pet is a pet”, not a family member: “My pet’s role in my family was not ideal. I felt that he was more than just a pet and I would caution everyone, not to get into that, just treat your pet as a pet”. Brenda mentioned that her seventh-grade daughter was too attached to the dog and has to limit the time she spends with the pet: “She wants to be with Molly all the time. Not only being with her, but sleeping as well. And I don’t like it, you know? I say, Love, everyone has to have their space. She also needs her space”.

*Worrying about sick pets.* When pets would fall ill, a third of parents (11 out of 31) witnessed how anxious family members, especially adolescents, would become. Marie described their dog not having an appetite: “So you know it’s natural that we get kind of worried, like is there something wrong, we’re not really sure. Does he have cancer? We get kind of freaked out”. Jessie also noted how her daughter was worried about their other dog losing his eyesight, and was “almost overly worried and [wanted] to put eyedrops in his eyes all the time”.

*Animal-related screen-time challenges.* Beyond remote learning, as adolescents search the internet for information related to their pets, the content they find is often useful and enjoyable but, in some cases, may be unpleasant, or even disturbing. Julie’s sixth-grade daughter, Rebecca, enjoyed exploring YouTube and TikTok videos for cute animals. Her older son, Jacob, who also used TikTok to watch animal videos, was fed a video of “one dog attacking and killing another dog” by the algorithm. Julie worried that Rebecca risked seeing similar disturbing, violent content even when searching unrelated animal videos.

### 3.3. Family Effects on Pet Wellbeing

While research more often focuses on the effect of pets on humans, most parents (24 out of 28 asked) believed that their children positively contributed to the well-being of their pets. Besides when there was a lack of awareness of pet well-being, parents mainly identified three scenarios where their child provided support: when pets faced stress and anxiety, dealt with illness, and during socialization with other animals.

*Lack of awareness for pet’s needs.* Occasionally, parents (4 of 28) believed that their child did not contribute to the well-being of their pet. Often, pets’ emotional needs were never previously considered, and parents were unable to give examples of caring about the pets’ needs. Parents thought their pets simply did not have needs similar to that of humans, with one parent stating “I’ve never really thought of him needing emotional support because he’s always so happy”. Though many parents often expected and relied on their pets to support the well-being of their child, this relationship was not necessarily reciprocated. Rarely, parents mentioned that their adolescents had a negative impact on pet well-being, such as yelling at a pet for barking or “pissing [the dog] off” due to limitations in their child’s capacity to have that social-emotional bond.

*Stress and anxiety.* Parents often (12 out of 24) described how their child supported the well-being of their pet by acknowledging and responding to their pets being stressed. For example, Cindy, found that her eighth-grade daughter was well attuned to “[paying] attention to [the] cues” of their dog, such as trembling. Stressful events also prompted care, as Rachel explained how her seventh-grade daughter Jenna would try to make their dog feel safe by providing encouragement when nervously crossing a slippery floor. Jennifer’s daughter Lucy provided similar verbal support for her dog who was too anxious to go into the pool.

*Illness.* Stressful medical events, including sickness and surgery, can also prompt youth to provide support. Marie recalls her son Paul being worried about the health of their dog:

[Paul] provided emotional support to the dog, so Milo has this remarkable habit of eating rabbit feces, which then he doesn’t eat for a day and a half, and is kind of like, mopey, like why? What’s wrong? Kind of seems like “don’t talk about food”, the dog is like, “I don’t want to talk about food”.

Similarly, Cindy explained how her daughter Lily expressed concern for the emotional well-being of her dog after surgery:

She would comfort him like, ‘Oh, what’s going on? She cares a lot, so she pays attention, she’s like, ‘Is Riley like, is he nervous, is he sick?...she comforted him a lot, and she will pay attention. And there was a time he had to go through a little tiny surgery, because he had something that they had to take off, it wasn’t a big deal, but you know, they treated him like a baby, like a sick baby, so they were up making sure he was fine and all that.

In parallel to how medical events prompted additional social and emotional attention from youth, children also provided physical care to their pets when they had health issues. Erin’s children “were like little nurses” providing water and food to their dog after surgery. Her children looked out for their dog’s well-being by continuing to lie on the floor and check on the pet over a gate. Another parent, Jessie, similarly mentioned how their dog’s gastritis prompted her eighth-grade daughter to gently hold and pet the dog to check on its well-being. On a different note, Rachel recalled that though her children refused to provide medical care for their cat’s hyperthyroidism, they were willing to accompany and pet their cat and keep them calm during veterinary appointments.

*Supporting socialization with other dogs.* In addition to providing direct emotional and physical support, Teresa explained how her daughters provide social support to encourage their pet dog’s socialization with other dogs. This parent remarked that following the pandemic, their dog lacked opportunities to socialize and would often “run behind the girl’s legs and be much more afraid of the bigger dogs”. This led their daughters to “help give her the courage to go socialize with other dogs”.

*Taking responsibility for the pet’s well-being*. Parents thought positively of how their children provided social, emotional and physical support to their pets. One parent, Kate, reflected fondly on how her 13-year-old son cared for their dog, as she found this level of affection to be unique given his age and gender identity and/or socialization. She found it “still kind of little-boy-ish” for her son to cuddle and talk sweet to their dog. Particularly, she admiringly noted how she would find her son repeating things she had said to their dog in a sweeter tone of voice than normal. Another parent, Cindy, was pleased that owning a dog encouraged her children to care for another being, instead of always being the center of attention. While the daughters had previously always had their needs and feelings taken care of, the dog provided them the opportunity to take care of somebody else:

Well there was something that I loved after we got the dog, after a while, and I felt like, finally, the girls care about somebody else, something else, for real, because before that everybody was taking care of them, you know, they were the most important thing. You know where everybody was covering their needs and their feelings and so now, there is something else that needs to be taken care of, and so I felt like, that shift from them not being the center of everything, but them taking care of somebody else, something else, it was really good for the whole family for them.

## 4. Discussion

Our findings contribute a greater understanding of the benefits and challenges of having a pet in the adolescent years from an underexplored perspective—the parent’s point of view. Most of our findings corroborated prior research self-reported from the child’s point of view that pets are a source of companionship (and are often viewed as a member of the family) [46], offering them a way to learn responsibility [47], and are a bridge to more comfortable social interactions with family members and friends [48,49]. In terms of emotional support, parents perceived that the pets not only provided their adolescent comfort when lonely but also when experiencing a range of negative emotions from anger to grief. Having empathy for another living being was crucial at this stage where parents observed that pets helped their adolescents become less self-centered.

Parents also reported the physical benefits that are typically explored in the case of adult dog ownership (i.e., walking them and getting exercise), which is consistent with prior research showing that youth with dogs are often more physically active (particularly with regard to walking) [50,51]. In the case of adolescents, parents often observed that spending time with the pet took adolescents away from their digital devices or, alternatively, was a way to feel less alone during homework or remote learning. A less commonly reported physical benefit was a physical sense of psychological safety and security that parents observed in their adolescents that is qualitatively different from a typical adult pet owner’s sense of safety in that dogs can protect their home and children when parents leave them all alone. This fundamentally changes a family system’s dynamic of relying on the pet–adolescent bond to keep them safe in case of danger.

An overlooked context in HAI research is the role that pets play in the use of screens, which in and of itself can serve as a source of social and emotional support [52]. As a social benefit, parents reported that pets often served as a topic to be actively featured on social media posts, which helped further online and offline social interactions. Prior research has demonstrated that adolescents tend to use their online spaces to maintain connection and intimacy with their offline friendships [53]. Adolescents’ online and offline worlds are psychologically connected, and they use these digital spaces to explore important developmental needs such as identity and exploring autonomy [54]. Finding like-minded online peers who share the same interests, including their mutual love of pets, can help reduce feelings of loneliness and increase a sense of belonging [10]. Parents observed that pets would often be a reason for their children to choose to reduce their screen time. Pets were also used for emotional comfort and therapeutic interactions (e.g., petting) during remote learning classes and when experiencing distressing content online. On the other hand, pets were also distracting and disruptive when it came to online learning contexts. Future quantitative research could explore when and for whom pets are conducive to reducing screen time and improving a sense of well-being when distressed online.

Parents expressed that barriers to successful adolescent–dog relationships can arise from behavior on the part of the child(ren) or the dog(s), and sometimes, but not always, cited parental actions (or inaction, such as allowing adolescents to fall behind on their pet responsibilities or not training the pet adequately) as the root of these behaviors. The challenges that parents faced, included ones that are faced by all pet owners and ones with children—the financial responsibilities, dread of the pet dying, and behavior that would be considered aggressive. The unique challenges that parents with adolescents face had to do with shared responsibility expectations and disappointments related to balancing pet duties amongst family members, who have shifting priorities as children grow into adolescence.

Another tension that is faced in adolescence is the jealousy over who the pet is most attached to, whether it be a sibling or a parent—the role of special confidant or loyal companion feels disrupted when there is a competing pet caregiver in the family. This may be particularly salient during the tween and early teen years when validation of one’s self-esteem may be crucial for their self-concept and social-emotional well-being [55]. Although many parents believed that the time and energy spent with pets outweighed more comforting benefits than harm, a few believed that their adolescent had grown too dependent on the pet, sometimes mentioning the pet as an unwelcome distraction from the real business of the day (e.g., schoolwork). In these scenarios, parents felt that adolescents were attached to pets in ways that were maladaptive, which, according to prior research [46], is a minority point of view. Another unique aspect of adolescence described by a minority of parents was that the timing of major developmental transitions during this period may be too tumultuous for introducing a pet into the household; however, the findings of a prior study that claimed that introducing a pet when a child is too young was also not recommended [37].

As HAI research most often focuses on the effects on humans, the effect that pet owners have on pets is another understudied area. In this study, parents who reported that their adolescents provided support to their pets found that it was often during times of stress, anxiety, or illness, as well as the encouragement of prosocial behaviors with other animals. Often adolescents were able to observe their pet needing attention and respond accordingly to address the pet’s well-being. Adolescents who saw their pet whimpering, approaching, or calling them demonstrated empathy by providing social, emotional, and physical support. Adolescents often provided social and emotional support in the form of encouraging or consoling words and companionship. Many parents also claimed that their child enjoyed providing physical support, often in the form of caretaking. Such willing participation in pet care by adolescents extends the literature that pet care behavior promotes attachment between children and their pets [36]. It is worth noting that while the support offered by adolescents is often well-intended, actions such as bathing cats may, in fact, be harmful to pets. Though such anthropomorphization of pets by adolescents is common, there is some consensus that such behavior should probably be avoided [56].

Overall, these findings underscore the importance and usefulness of taking a developmental systems approach to understanding human–animal interaction in the family setting. Many of the types of pet–adolescent interactions reported by parents involved dynamic relationships across the whole family system. For example, parents noted their role in shaping the degree to which youth were responsible for the care of the pet, as well as parental actions being a key factor in successfully or unsuccessfully managing behavior challenges with animals. Parent reflections on jealousy regarding attention from the pets further emphasize how companion animals can influence, and are influenced by, interpersonal dynamics within the family. Other signs of pets being integral to a family’s evolving ecosystem during adolescence included parents’ reflections on their adolescents’ developing needs in the realm of gaining autonomy (e.g., leaving them home alone with a dog) and seeking identity and connection with others online. In order to fully understand the role of companion animals in adolescent development, researchers need to use methodological approaches that assess all integrated aspects of the developmental ecosystem.

### Limitations

Although we aimed to recruit from the more diverse U.S. survey sample, the parents who self-selected to be interviewed were not as racially/ethnically or socioeconomically diverse as the larger U.S. sample. These findings may be somewhat limited to the Northeast U.S. geographic region, or more generalizable to families who live primarily in middle-higher income suburban areas. One of the unique contributions of our study is to bring parent/guardian perspectives about the role of pets in their household into the HAI field, however, the perspectives from their children’s point of view were not included in the current study. We also primarily interviewed mothers, people who had the free time to speak with us for roughly an hour with us during the workweek, and people with both reliable internet access and internet literacy. A review of prior research [20] has identified limited studies of pets other than dogs due to their unique potential for social interactions compared to other pet types. Although our sample also focused more frequently on dog contributions, we were able to highlight the contributions of other pets to adolescent social, physical, and emotional well-being by capitalizing on qualitative methods, given that the typical unevenness of pet type distribution in most survey studies makes this comparison difficult. Given the scope of this paper, the focus was only on the efforts of pet owners to support their pets’ well-being, as opposed to the effects of such efforts. As the relationship between the pet owners and their pets may be reciprocal, future research could focus on the results of social, emotional and physical support for pets.

Regarding the potential limitation of generalizability of this higher-income parental sample, there was no indication that the interview sample had a higher socioeconomic level than the larger study population (in fact, had a higher percentage of lower-income households than the larger survey sample). Reported income level did not seem to influence whether or not a parent volunteered to participate in an interview. However, we are likely not capturing the lowest income families’ experiences which may have a unique set of challenges compared to higher-income families. Because we did not recruit any families with non-binary youth, these findings may not be generalizable to all types of adolescent gender identity and pet interactions. The data were also collected during the COVID-19 pandemic, a period that saw significant changes in the routines of adolescents and their families [5,6]. While it is important to study the relationships between pets and adolescents in the unique contexts of lockdown and remote learning, the time frame of data collection limits the generalizability of our findings.

## 5. Conclusions and Future Directions

There is a growing body of research that demonstrates the benefits of companion animals for improving well-being; however, the research has also found null effects, highlighting recruitment, analytical and publication bias around the positive effects of companion animals. In addition, studies are conducted primarily on child or adult populations without the necessary attention on diverse families, types of human–animal interactions, under what conditions, or what characteristics of individuals in vulnerable developmental stages are associated with well-being. In addition, there is a dearth of in-depth qualitative research on the well-being effects of family pets on adolescence, including social support, socioemotional well-being, family relations, and reduced screen time. Little is known about the purpose of pets in each family’s lives and how parents/guardians overtly and covertly socialize their children in attitudes toward animals, caretaking, companionship, and social-emotional resilience. We have documented that parents often perceive that the benefits of pets outweigh challenges, with many contextual factors in determining the right fit between the child/pet/family system, the timing of pets and age of adolescents, and the goals that each family has for the pet companionship.

The results from this study indicate that parent/guardian perspectives can provide important insight into youth–pet relationships in daily life. More research is needed on the potential role of parents as an informant on the pet–adolescent relationship within families, including triangulation with youth-report data. Better understanding the circumstances under which companion animals can be beneficial for youth and the factors that promote thriving among animals will allow for evidence-based recommendations for families and both human and veterinary practitioners.

## Figures and Tables

**Table 1:** Qualitative themes of pet influences on adolescents.

	Benefits	Challenges
I. Social	Pet as a companion	Pet as a distraction
	Learning about responsibility	Family tension regarding shared responsibilities
	Building empathy skills	Jealousy issues
	Bonding with family and friends offline	
	Bonding through social media	Screens pull attention away from pets
II. Physical	Physical activity displacing screen time	Pet growing bigger
	Bonding with family during exercise	Unable to travel
	Sense of physical/psychological safety	Problematic animal behaviors
III. Emotional	Managing anxiety	Financial burden
	Regulating emotions offline (anger, grief, loneliness)	Overly attached
	Regulating emotions during digital media use	Worrying about sick pets

## Data Availability

De-identified qualitative dataset available upon request.
